# Molecular Piracy: Redirection of Bacteriophage Capsid Assembly by Mobile Genetic Elements

**DOI:** 10.3390/v11111003

**Published:** 2019-10-31

**Authors:** Terje Dokland

**Affiliations:** Department of Microbiology, University of Alabama at Birmingham, Birmingham, AL 35242, USA; dokland@uab.edu

**Keywords:** capsid assembly, *Staphylococcus aureus* pathogenicity island, phage-inducible chromosomal islands, transduction

## Abstract

Horizontal transfer of mobile genetic elements (MGEs) is a key aspect of the evolution of bacterial pathogens. Transduction by bacteriophages is especially important in this process. Bacteriophages—which assemble a machinery for efficient encapsidation and transfer of genetic material—often transfer MGEs and other chromosomal DNA in a more-or-less nonspecific low-frequency process known as generalized transduction. However, some MGEs have evolved highly specific mechanisms to take advantage of bacteriophages for their own propagation and high-frequency transfer while strongly interfering with phage production—“molecular piracy”. These mechanisms include the ability to sense the presence of a phage entering lytic growth, specific recognition and packaging of MGE genomes into phage capsids, and the redirection of the phage assembly pathway to form capsids with a size more appropriate for the size of the MGE. This review focuses on the process of assembly redirection, which has evolved convergently in many different MGEs from across the bacterial universe. The diverse mechanisms that exist suggest that size redirection is an evolutionarily advantageous strategy for many MGEs.

## 1. Introduction

Horizontal evolution through the dispersal of mobile genetic elements (MGEs) such as plasmids, bacteriophages and chromosomal islands is a key element in bacterial evolution and the development of bacterial pathogenicity [1,2]. MGEs may be transferred between bacteria by transformation, transduction or conjugation. However, for many bacteria, such as *Staphylococcus aureus*, that are not naturally competent for transformation and do not commonly undergo conjugation, transduction by bacteriophages is the main mechanism of horizontal gene transfer (HGT) [3,4].

**Figure 1:**
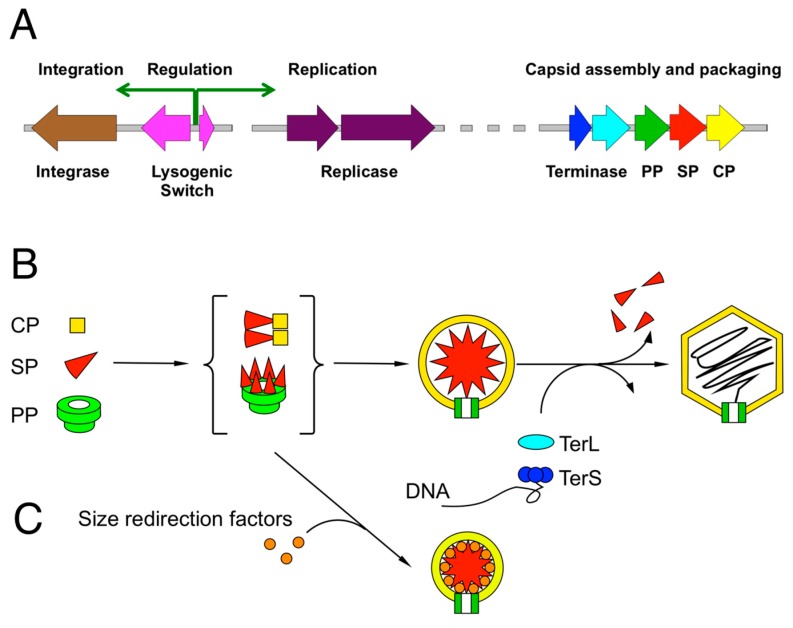
The bacteriophages of the *Caudovirales*. (**A**) Typical modules found in *Caudovirales* genomes, including integration, replication and capsid assembly/DNA packaging modules. (**B**) General assembly pathway for bacteriophages. Precursor procapsids are formed from capsid protein (CP), scaffolding protein (SP) and portal protein (PP) through poorly characterized CP-SP and/or PP-SP intermediates (curly brackets). In some systems, the SP functionality is fused to CP. Procapsids are packaged with DNA by the terminase complex (TerL and TerS), leading to capsid expansion and removal of SP. (**C**) Size redirection factors encoded by “pirate” MGEs shunt the capsid assembly pathway to small capsid formation.

The bacteriophages involved in HGT are the tailed bacteriophages with double-stranded DNA genomes that belong to the order *Caudovirales*, which is divided into three families based on their tail structure: *Myoviridae* (contractile tails), *Siphoviridae* (long, flexuous tails) and *Podoviridae* (short tails) [5]. These viruses are considered to be ancient entities that are all deeply related, as evidenced by the similarities in their genomic organization and in the presence of many conserved structural elements that are unique to bacteriophages (Figure 1A); that is to say, the *Caudovirales* represent a monophyletic group [6].

During the lytic cycle, many bacteriophages are capable of *generalized transduction*, during which they pick up and package chromosomal or plasmid DNA from the host at a low frequency [7]. Many members of the *Caudovirales*—the *temperate* phages—are capable of undergoing *lysogeny*, during which their genomes are integrated as *prophages* into their host genomes. If these prophages carry genes that are advantageous to their hosts, e.g., encoding virulence or resistance factors, they may become established in the bacterial population. Prophages may excise and re-enter the lytic cycle in response to environmental cues, such as UV or DNA-damaging chemicals. Temperate phages sometimes pick up adjacent genetic material during the process of excision (usually referred to as *specialized transduction*). A related process termed *lateral transduction* involves in situ replication and packaging and is capable of packaging DNA several thousands of base pairs downstream of the prophage [7]. In many cases, prophages lose their ability to excise and become cryptic or defective phages, permanently entombed within their host genomes [8].

However, some MGEs have developed a more intimate relationship to specific bacteriophages, known as “helpers”. In this case, the MGE is packaged by the phage at a much higher frequency than that expected by generalized transduction. “Helper” is maybe not the best word, since the phage is an unwilling victim in this process, whose own propagation is severely suppressed. We have coined the term “molecular piracy” to describe the process by which these MGEs usurp the phage life cycle [9]. These molecular pirates have evolved several mechanisms to interfere with phage production, including the exploitation of the phages’ early lytic proteins to initiate their own excision and replication, transcriptional trans-activation and suppression, selective packaging of their own genomes, and re-direction of the phage assembly pathway to form capsids that are appropriate for their own smaller genomes [9]. There is a strong selective pressure on the phage to evolve resistance to this piracy, and helpers and pirates are therefore engaged in a constant co-evolutionary battle. This review will focus on one particular aspect of this piracy, namely the redirection of the phage assembly process to form capsids that are too small to package complete phage genomes. Different MGEs have evolved a variety of mechanisms to accomplish this task, indicating that capsid size redirection offers evolutionary advantages to the pirate elements.

## 2. Review of Capsid Assembly and DNA Packaging in the *Caudovirales*

Since the molecular pirates are intimately dependent on structural proteins provided by their helpers and on their helpers’ assembly process, it is necessary to review the general assembly pathway of bacteriophages. All *Caudovirales* members assemble their virions by a similar process (Figure 1B): Empty precursor capsids (*procapsids*) are made from a major capsid protein (CP), a scaffolding protein (SP) that acts as a chaperone for the assembly process, and a portal protein (PP, in some systems called the “connector”) that will form the entry and exit portal for the DNA. The portal also serves as an attachment point for the tail and related structures. Cleavage of structural proteins by a phage- or host-encoded protease is common, but not universal. In some cases, the SP is part of the CP (as an N-terminal δ domain in HK97 and related phages) or the protease (as in phage P2). Many phages package additional minor capsid proteins that serve various roles but are not typically required for capsid assembly per se. DNA is packaged into these procapsids through the portal by the terminase complex, which consists of a small subunit (TerS) that is responsible for recognizing the DNA, and a large subunit (TerL) that carries out the DNA packaging in an ATP-driven process [10]. In most cases, the substrate for DNA packaging is linear, concatemeric DNA generated by rolling-circle replication, but in some cases, closed circular or linear monomeric DNA is preferred. Packaging starts with the recognition of a *pac* or *cos* sequence in the genome. When the capsid is full—and, in the case of *cos* site packaging phages, when a second *cos* site is recognized—the DNA is cleaved by the TerL nuclease domain, and packaging continues on another capsid. Tails are assembled via a separate pathway and attached to the capsids after packaging.

All members of the *Caudovirales* studied to date share a similar capsid protein (CP) structure, known as the “phage capsid fold” or “HK97 fold”, after the first high-resolution phage capsid structure that was determined [11]. Some phages, such as T4 and P22, have additional domains inserted into the basic HK97 fold [12,13]. The capsid fold is remarkably adaptable and exists in both isometric and prolate capsids of a wide range of sizes and architectures (T-numbers) [14,15]. (Icosahedral capsid structure is described by its *T*-number, which represents how the 20 triangular faces of the icosahedron are divided into *T* smaller sub-triangles. It generally also reflects the number of CP subunits that comprises the capsids (60*T*). According to the rules of quasi-equivalence, certain numbers are preferred, following the formula *T* = h^2^ + hk + k^2^, leading to numbers 1, 3, 4, 7, 9, 12, 13 … However, other numbers are possible, most notably 2. Prolate capsids are described by two numbers, *T*_end_ and *T*_mid_ (or Q), referring to the end caps and the cylindrical midsection, respectively.) In various systems, mutants are known in CP, SP and PP that lead to formation of capsids of a different size, both smaller [12,16,17,18] and larger (e.g., T4 “giant” heads [19]) than normal. Control. mechanisms are required to curtail this flexibility to produce only capsids of the right size [20], in the absence of which capsid proteins often self-assemble into aberrant structures, called “monsters” or “crapsids”. The main protein involved in such control. is the SP (or the equivalent δ domain) [21], but the PP and other internal capsid proteins may also be involved [22]. In T4, for example, correct capsid size is dependent on the non-essential Alt protein [22]. Phage P1 naturally forms closed, viable capsids of several sizes [23], but this is also affected by mutations in the Dar antirestriction proteins [24]. Host factors, such as GroEL for T4 and lambda [25] and the Prp protease for *S. aureus* phage 80α [26,27] also play important roles.

Since the SPs are generally removed during capsid maturation and tend to be disordered and flexible, few SP structures are still known. The most complete structure of an SP is gp7 from the prolate *Bacillus* podovirus Φ29, which is a dimer made from an all-helical protein that forms a hairpin [28]. Partial structures are available for the SPs of *Salmonella* phage P22 [29] and *S. aureus* phage 80α (gp46) [30]. It appears to be a common feature among these proteins that they are almost entirely α-helical with a propensity for coiled-coil formation.

Pirate MGEs take advantage of this malleability of capsid proteins to redirect the helper phages’ assembly pathways to produce capsids with a size more suitable for their own, generally smaller genomes, to the detriment of their helpers, which are thereby unable to complete their own packaging.

## 3. The P2/P4 Paradigm

The P2/P4 system represents the classic example of molecular piracy. P2 was first described by Joe Bertani in the early 1950s [31]. It is a myovirus with a somewhat smaller-than-typical genome size of 33 kbp packaged in a 60 nm, *T* = 7 isometric capsid [32]. P2-like phages are common in *Escherichia coli* and related bacteria [33].

**Figure 2:**
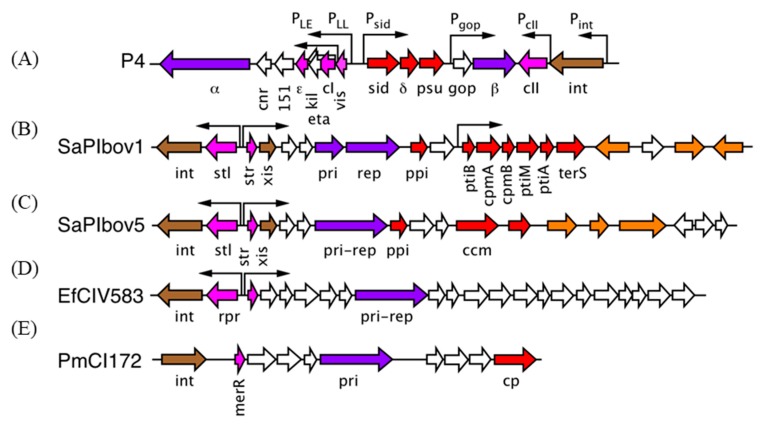
Genomes of pirate elements. (**A**) P4, (**B**) SaPIbov1, (**C**) SaPIbov5, (**D**) EfCIV583, (**E**) PmCI172. Color coding: genes involved in integration (*int*) and excision (*xis*), brown; replication-related genes (primase, replicase), purple; transcriptional regulators (repressors, activators), pink; genes demonstrated or presumed to be involved in helper exploitation (including capsid size redirection), red; virulence factor genes, orange. Other genes, white.

“Satellite bacteriophage” P4 was discovered by Erich Six as an MGE that depended on P2 for its mobilization [34]. Upon closer inspection, P4 is not really a phage at all, but an integrative plasmid (phasmid or phagemid), an MGE that can replicate as a plasmid or integrate into the host genome [35,36] (Figure 2). However, P4 lacks genes encoding major structural proteins and is unable to form infectious particles on its own. When a cell harboring P4 is infected with P2 (or a related helper phage), P4 becomes packaged into phage particles made mostly from P2-encoded structural proteins [37]. However, the resulting P4 capsids are smaller (*T* = 4) than those normally made by P2 (*T* = 7) [38,39]. This size redirection function was found to reside in the *sid* (*si*ze *d*etermination) gene of P4 [40,41].

**Figure 3:**
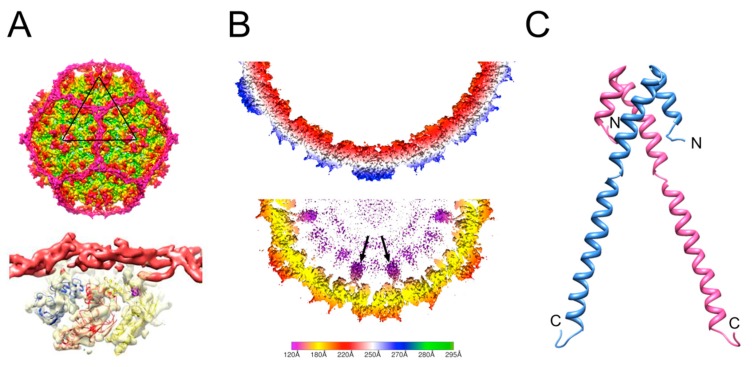
Size redirection by P4 and type 1 SaPIs. (**A**) Isosurface representation of the P4 procapsid reconstruction (top) showing the dodecahedral cage made by Sid (magenta). The bottom image shows the Sid density (red) as it interacts with the CP hexamer underneath (yellow surface; three copies of CP are modeled into the density). (**B**) Slice through the three-dimensional reconstructions of 80α (top) and SaPI1 (bottom) procapsids, scaled radially according to the color bar (radius in Å). The internal protrusions corresponding to CpmB (purple) are indicated by arrows. (**C**) Ribbon representation of the SaPI1 CpmB dimer, made as a composite between the CpmB NMR structure (PDB ID: 2L8T) and the SaPI1 cryo-EM structure (PDB ID: 6B23). N- and C-termini are labeled.

Cryo-EM studies showed that Sid forms a dodecahedral cage around the P4 procapsid (Figure 3A) that makes trimeric connections at the threefold axes and connects with hexamers formed by the gpN capsid protein at the twofold axes [39,42]. Sid itself is entirely α-helical and elongated, with a high propensity for coiled-coil formation, but its exact fold has not yet been determined. The Sid cage restricts the number of gpN hexamers that can be fitted onto the procapsid, resulting in a *T* = 4 lattice, although occasionally, a few capsids of aberrant shapes and sizes may be formed. Small capsids can form from gpN and Sid alone, but the gpO scaffolding protein is required for viability, presumably because it serves other essential functions, including portal incorporation and maturation cleavage of structural proteins [43].

## 4. The *Staphylococcus aureus* Pathogenicity Islands (SaPIs)

*Staphylococcus aureus* pathogenicity islands (SaPIs) are a large family of chromosomal islands, typically around 14 kb in size, that share a common structure: an integrase at one end of the genome, followed by a repressor gene expressed in one direction and a set of genes that includes a replication module expressed in the opposite direction (SaPIbov1, Figure 2) [44,45,46,47]. The repressor and replication genes are under control. of a pair of opposing promoters that resemble a phage lytic/lysogenic switch. Towards the right end of the genome is a “helper exploitation” module encoding genes involved in phage interactions. SaPIs were first identified as the carrier of the *tst* gene that encodes the superantigen toxic shock syndrome toxin (TSST-1) [48]. Other SaPIs often encode a variety of superantigen toxins, but other virulence factors, such as adhesins and coagulases, and antibiotic resistance factors (in which case it is most correctly termed a SaRI) are also found. Closely related islands are found in other staphylococci, in streptococci and listeriae, and collectively referred to as “phage-related chromosomal islands” or PRCIs [45,49].

Type 1 SaPIs encode a helper exploitation cluster that includes two genes called *cpmA* and *cpmB* [50,51] (Figure 2). *cpmB* was found to encode a dimeric α-helical protein with a remarkable similarity to the SP of phage Φ29 [28,51] (Figure 3C). The C-terminal part of CpmB is highly conserved between SaPIs and related elements in other species and includes an RIIK motif that is also present at the C-terminus of the SP of 80α and related phages [51]. Cryo-EM analysis revealed that CpmB binds to the 80α CP in the same site as the cognate SP using the conserved C-terminal sequence and that the binding alters the angle between capsomers that thereby changes the curvature of the shell and consequently the size from *T* = 7 to *T* = 4 [30] (Figure 3B). The similarity in capsid binding between SP and CpmB suggests that the two proteins compete for the binding site on CP. By using the same binding site as the cognate SP, the phages are thus unable to escape the size redirection.

The role of CpmA in this process is still mysterious. CpmA is required for size redirection, and if only CpmB is present, normal large capsids are formed. However, if only CpmA is present, assembly tends to be aberrant [50,52]. The current thinking is that CpmA binds to SP and removes it from the CP and is required to allow CpmB access to CP [30]. This would explain why CpmA alone is detrimental to assembly. However, this model has yet to be tested experimentally.

The *cpmA* and *cpmB* genes are highly conserved and always come together. However, the proteins are not required for SaPI mobilization. In the absence of size redirection, SaPIs are packaged equally well into large capsids as concatemers and phage propagation is repressed through other mechanisms [9,46,47]. Type 1 SaPIs encode a TerS subunit that recognizes their own unique *pac* site [53] and a protein, Ppi, that blocks phage DNA packaging [54]. Nevertheless, the conservation of the *cpmAB* genes indicates that size redirection is evolutionarily advantageous to the SaPIs in their natural environment.

## 5. A Different Type of SaPI

**Figure 4:**
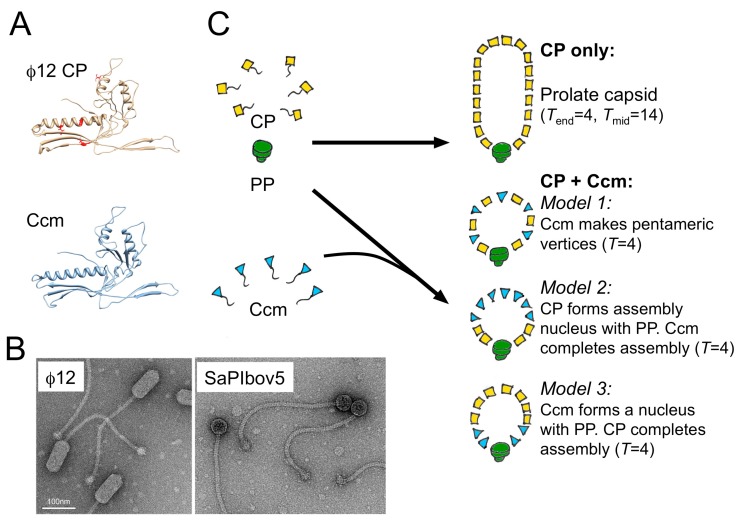
Size redirection by SaPIbov5. (**A**) Ribbon diagram of the I-TASSER models of the Φ12 CP (yellow) and SaPIbov5 Ccm (blue). The sir mutations in CP are indicated in red. (**B**) Negative stain electron micrographs of Φ12 virions and the transducing particles formed in the presence of SaPIbov5. (**C**) Three models for size redirection by Ccm: In model 1, Ccm forms pentamers at capsid vertices. In model 2, PP and CP form a nucleus, while Ccm completes the shell. In model 3, PP and Ccm form the nucleus and CP completes the shell.

The original identification of SaPIs included a divergent group (type 2) that shared the integration, repressor and replication modules of SaPI1 and its relatives but lacked the typical helper exploitation module found in SaPI1 [55,56]. Instead, a different kind of cluster was observed [57] (SaPIbov5, Figure 2). These type 2 SaPIs can be packaged by 80α and similar phages but do not change the size of the capsid as they lack CpmA and CpmB. However, at least one member of this group, SaPIbov5, can also be mobilized by prolate, *cos*-packaging phages such as Φ12 and ΦSLT. Strikingly, in this case, the shape and size of the helper is changed from its usual prolate form to an isometric capsid [57] (Figure 4).

This size redirection was found to be dependent on a gene present in the distinct helper exploitation cluster of SaPIbov5 that was named *ccm*, for *cos*-phage capsid morphogenesis [57]. The Ccm protein has a phage capsid-like fold with 23% sequence identity to that of its Φ12 helper, suggesting that it evolved from it by duplication. It is still unknown how Ccm changes the size of its capsid, although it is known that it gets incorporated into (small) capsids and is thought to replace CP at some positions. However, the Φ12 CP is also required for SaPIbov5 capsid formation. Mutants in CP (*sir* mutations) render Φ12 resistant to the SaPIbov5-indced size redirection.

We recently determined that the prolate Φ12 capsid has *T*_end_ = 4 and *T*_mid_ = 14 architecture, while the isometric SaPIbov5 capsids are *T* = 4, suggesting that size redirection consists of removal of the cylindrical midsection of the prolate helper (Hawkins, Kizziah and Dokland, in preparation). How does Ccm accomplish this? At least three possible models can be outlined (Figure 4): (Model 1) Ccm forms pentamers. An isometric capsid has a higher pentamer-to-hexamer ratio than a prolate one, so it would be reasonable to imagine that the assembly process can be biased toward small (isometric) capsids by introducing more pentamers into the system. This model is consistent with the observation of small oligomers upon Ccm expression in *E. coli* (unpublished data). However, if this model were correct, one might also expect to observe prolate capsids of intermediate size, which are not typically seen. (Model 2) CP forms an assembly nucleus around the portal, with Ccm completing the shell. In this model, the assumption is that Ccm is unable to nucleate on its own, but once initiated, will assemble isometric shells. (Model 3) Ccm forms a size-determining nucleus with the portal protein, onto which the Φ12 CP is required to complete the shell. While this model is also consistent with observations, it is unclear what kind of mechanism the portal-Ccm nucleus would employ to induce a specific size on CP. Clearly, more structural and functional data is still needed to distinguish these different models.

## 6. The Families of Phage-Induced Chromosomal Islands (PICIs)

After the discovery of SaPIs, the Penadés lab initiated a bioinformatics search for elements in other hosts that share the same kinds of characteristics as SaPIs, *viz.* an integrase, repressor and replication modules, and the presence of *att* sites. Several such elements were identified in Firmicutes, including *Enterococcus*, *Lactococcus* and *Streptococcus* [58]. As a group, all these elements were designated “Phage-Inducible Chromosomal Islands” or PICIs (equivalent to PRCIs, mentioned earlier). At least one of these islands, EfCIV583 (Figure 2), could be mobilized by mitomycin C induction of a prophage present in the same strain (*Enterococcus faecalis* V583), resulting in packaging of small size DNA, indicating formation of small capsids [58]. EfCIV583 does not contain any recognizable capsid- or scaffolding-related genes (Figure 2), so how this size redirection is accomplished is still unknown.

A functionally similar, but genetically distinct group of PICIs was subsequently identified in Gram-negative organisms, including *Escherichia coli* and *Pasteurella multocida* [59]. For one of these PICIs, PmCI172 (Figure 2), small, isometric capsids were observed by EM after induction with mitomycin C. The resident prophage in *P. multocida* strain 172 is a Mu-like myovirus with an isometric capsid of a size expected of *T* = 7 architecture, while the PmCI172 capsid size is consistent with *T* = 4, commensurate with the sizes of their respective genomes. This may suggest that capsid redirection proceeds by the same mechanism as that of P2/P4 or 80α/SaPI1. However, no genes corresponding to either a P4-like Sid protein or SaPI1-like CpmAB proteins were identified in PmCI172. This PICI does encode a capsid-like protein (Figure 2) and it is tempting to speculate that this protein is involved in the size redirection mechanism, similarly to Ccm of SaPIbov5. However, the fact that the change is from a large to a small isometric capsid—rather than prolate to isometric—suggests that the size redirection proceeds by yet another still undetermined mechanism. A similar PICI present in *E. coli* (EcCICFT073) also encodes a capsid protein homolog but appears to be packaged into capsids of normal (large) size, at least when induced by phage lambda [60].

## 7. Evolution of Molecular Piracy

As the preceding examples illustrate, capsid size redirection is a common theme among the molecular pirates. However, although the capsids of all tailed dsDNA phages are structurally related, the mechanisms for altering the natural assembly pathway of these capsids are diverse, presumably reflecting convergent evolution by several different types of MGEs.

**Figure 5:**
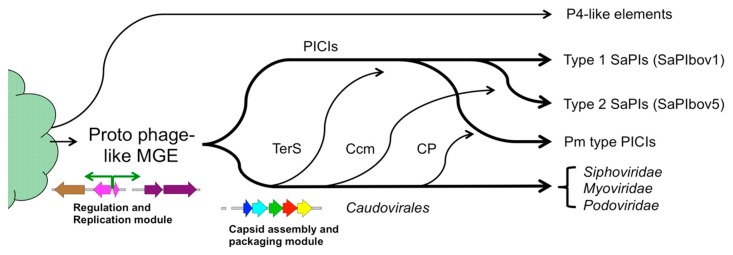
Model for the evolution of molecular piracy by size redirection. The phages and PICIs are ancient elements that originated from a proto-phage and diverged early, separated by the ability of phages to form capsids. The various families of PICIs form separate branches on the evolutionary tree that acquired phage-like functions by horizontal evolution at different times. P4-like elements evolved separately from PICIs from a distinct origin.

The phages of the *Caudovirales* are assumed have arisen from a common ancestor. Similarities between phages and PICIs, specifically the similar integrase, repressor and replication modules, suggest that these too arose from the same proto-phage-like ancestor (Figure 5). Apparently, the PICIs either never acquired the structural gene modules of phages and thus the ability to form virions or lost them early in their evolutionary history. Either way, the lack of structural genes left them dependent on helper phages for their own mobilization.

The variety of capsid re-direction mechanisms suggests that these arose at different times by horizontal acquisition of phage-like structural genes, in particular those encoding capsid and scaffolding proteins (Figure 5). Once established, the PICIs harboring specific size redirection apparatuses would have formed distinct branches on the evolutionary tree. P4-like elements, on the other hand, appear to have arisen independently from phages and PICIs (Figure 5), most likely being more closely related to plasmids [35]. These elements have also acquired the ability to redirect helper capsid assembly but appear to have done so by an independent mechanism, since there are no obviously Sid-like proteins found in phages.

Why did size redirection mechanisms evolve? The small capsid size is obviously more appropriate for the typically smaller pirate genomes. (Larger elements, like SaPIbov2, are by necessity packaged into large capsids.) However, in the type 1 SaPIs, transducing titers are no different whether the SaPI genomes are packaged into large or small capsids [50]. In headful packagers, DNA packaging proceeds from the concatemeric DNA substrate until the capsid is full, and as long as the capsid has at least one full-length genome, it will be fully infectious. In the case of *cos* site packagers, the situation is somewhat more complicated, since in order to complete packaging successfully, there has to be a *cos* site in the proximity of the terminase complex when the capsid is full. Three P4 genomes can be packaged into P2-sized capsids, but the efficiency is low, presumably because the resulting genome size is not optimal for the capsid [40]. For SaPIbov5, transducing titers were reduced when the genome size was made 10% larger or 18% smaller, and packaging into small capsids was defective [57]. Presumably, concatemers of two or three of these constructs were more efficiently packaged into large capsids. It is likely that the size requirements of *cos* site packaging provides an additional constraint on the available genome sizes for *cos* packaging MGEs, which could favor the evolution of size redirection mechanisms.

By making small capsids, phage production is strongly curtailed. This suppression is evolutionarily advantageous, presumably by limiting the amount of circulating phage that—if allowed to grow unrestrained—could wipe out the entire bacterial population harboring the pirate elements. On the other hand, it would not be advantageous to eliminate the phages completely, since they provide the means for pirate elements to spread. Thus, an evolutionary balance is reached. For P4, size redirection is the only way to suppress phage production, and much more phage is produced in a *sid* mutant that does nost make small capsids [40]. Similarly, Φ12 titers were 10^6^–10^7^ higher in the presence of a *ccm* mutant than with wildtype SaPIbov5 [57]. For the type 1 SaPIs, however, phage production is strongly suppressed even when the size redirection genes are deleted [50]. This is because SaPIs interfere with phage production via other mechanisms, including direct suppression of phage DNA packaging and transcriptional trans-regulation of phage late gene expression [9,46]. However, the fact that these mechanisms are almost universal and highly conserved within groups of MGEs suggests that size redirection offers an evolutionary advantage in the natural environment of the hosts, where one must assume that multiple strain variants and numerous MGEs co-exist, leading to a complex evolutionary inter-relationship.
